# Incidence of *Wolbachia* in aquatic insects

**DOI:** 10.1002/ece3.2742

**Published:** 2017-01-24

**Authors:** Eric J. Sazama, Michael J. Bosch, Carmelita S. Shouldis, Scot P. Ouellette, Jeff S. Wesner

**Affiliations:** ^1^ Department of BiologyUniversity of South DakotaVermillionSDUSA; ^2^ Division of Basic Biomedical SciencesSanford School of MedicineUniversity of South DakotaVermillionSDUSA

**Keywords:** aquatic insects, endosymbiont, Missouri River, *Wolbachia*

## Abstract

*Wolbachia* is a genus of intracellular bacteria typically found within the reproductive systems of insects that manipulates those systems of their hosts. While current estimates of *Wolbachia* incidence suggest that it infects approximately half of all arthropod species, these estimates are based almost entirely on terrestrial insects. No systematic survey of *Wolbachia* in aquatic insects has been performed. To estimate *Wolbachia* incidence among aquatic insect species, we combined field‐collected samples from the Missouri River (251 samples from 58 species) with a global database from previously published surveys. The final database contained 5,598 samples of 2,687 total species (228 aquatic and 2,459 terrestrial). We estimate that 52% (95% CrIs: 44%–60%) of aquatic insect species carry *Wolbachia*, compared to 60% (58%–63%) of terrestrial insects. Among aquatic insects, infected orders included Odonata, Coleoptera, Trichoptera, Ephemeroptera, Diptera, Hemiptera, and Plecoptera. Incidence was highest within aquatic Diptera and Hemiptera (69%), Odonata (50%), and Coleoptera (53%), and was lowest within Ephemeroptera (13%). These results indicate that *Wolbachia* is common among aquatic insects, but incidence varies widely across orders and is especially uncertain in those orders with low sample sizes such as Ephemeroptera, Plecoptera, and Trichoptera.

## 1 Introduction

*Wolbachia* is a genus of bacteria found within the tissues of several groups of arthropods (Pietri, DeBruhl, & Sullivan, 2016; Werren, Baldo, & Clark, 2008). They typically infect the reproductive tissues of insects where they can manipulate reproduction of their hosts to enhance the vertical transmission of *Wolbachia* from mother to offspring (Werren et al., 2008). Recent estimates for *Wolbachia* placed infection rates among arthropod species at 40%, 52%, and 66% (Hilgenboecker, Hammerstein, Schlattmann, Telschow, & Werren, 2008; Weinert, Araujo‐Jnr, Ahmed, & Welch, 2015; Zug & Hammerstein, 2012). These estimates show that *Wolbachia* is a common symbiont within arthropods, making it arguably the most abundant intracellular bacteria (Werren et al., 2008).

Despite the high incidence among arthropods in general, little research exists on the incidence of *Wolbachia* in aquatic insects, defined as insects whose larval stages are in freshwater aquatic environments. For example, using data from a recent global meta‐analysis (Hilgenboecker et al., 2008), we estimate that less than 5% of tested insect species have aquatic stages in their life‐history. Those surveys appear to only include the winged stages of some aquatic insects found in terrestrial areas, sampled haphazardly as part of broader surveys focused on terrestrial arthropods. Other studies have tested members of the order Odonata and aquatic Coleoptera (Sontowski, Bernhard, Bleidorn, Schlegel, & Gerth, 2015; Thipaksorn, Jamnongluk, & Kittayapong, 2003), but very few studies have investigated other major aquatic taxa such as Ephemeroptera, Trichoptera, and Plecoptera (but see Werren & Windsor, 2000; Prakash & Puttaraju, 2007; Yun et al., 2014). These types of surveys are important to understand the global infection frequency of *Wolbachia* so that future research on the potential impact of these bacteria on arthropod populations can be assessed. While these studies provide estimates of infection within specific groups, to our knowledge no systematic survey of *Wolbachia* incidence within aquatic insects has been performed.

Here, we estimate the incidence of *Wolbachia* in aquatic and terrestrial insects using data from our own field collections along with previously published databases (Sontowski et al., 2015; Weinert et al., 2015; Wiwatanaratanabutr & Zhang, 2016). We present estimates of *Wolbachia* incidence for aquatic and terrestrial insects as a whole, and also for individual orders of major aquatic insects. We suggest that the incidence of *Wolbachia* in aquatic insects is comparable to that in terrestrial species.

## 2 Materials and Methods

### 2.1 Specimen collection

Insects were collected in the summer (May–August) of 2014 and 2015 from eight nearby rivers and streams connected to the Missouri National Recreational River as well as the Missouri River itself (Table S1). Larval insects sampled from the substrate were taken using a D‐frame dip net at each site. Adults were captured using three floating emergence traps with an area of 0.36 m^2^ each, set for 3 days per sampling trip. Different habitats (e.g., debris, riprap, shoreline) were sampled when available to increase taxonomic representation. Traps were set once per week during the sampling period. Benthic samples were taken once when emergence traps were set and again when traps were retrieved. Adults were aspirated from the emergence traps at the end of the sampling period. Upon collection, samples were preserved in 95% ethanol on site and stored at −20°C at the University of South Dakota. Specimens were sorted and identified to the lowest possible taxonomic level or by morphological characteristics (species and morphospecies) and stored until DNA extraction.

### 2.2 DNA extraction

DNA extraction was performed using a DNeasy Blood and Tissue kit (QIAGEN). Selected portions of each specimen were used to extract the DNA. Extraction from larger specimens involved taking only the abdomen, whereas smaller specimens were decapitated to prevent inhibitors from interfering with PCR (Beckmann & Fallon, 2012). Upon complete extraction, samples were analyzed using a NanoDrop 2000 Spectrophotometer (Thermo Scientific) to obtain DNA concentrations. PCR was performed on approximately 100 ng of genomic DNA from the samples using wspF (5′ GTCCAATARSTGATGARGAAAC 3′) and wspR primers (5′ CYGCACCAAYAGYRCTRTAAA 3′), yielding a product approximately 600 bp in size. Methods were adapted according to previously established protocols (Baldo et al., 2006). A thermocycler was set for the following conditions: denaturation at 95°C for 5 min, followed by 45 cycles of denaturation at 95°C for 30 s, annealing at 59°C for 45 s, elongation at 72°C for 90 s and a final elongation step at 72°C for 10 min. Universal 16S rDNA primers used were 341F (5′ CCTACGGGAGGCAGCAG 3′) and 534R (5′ ATTACCGCGGCTGCTGGC 3′) with the following conditions: 95°C for 5 min, followed by 40 cycles of denaturation at 95°C for 30 s, annealing at 54°C for 30 s, elongation at 72°C for 60 s and a final elongation step at 72°C for 10 min. A 2% agarose gel with ethidium bromide was used to visualize results of the PCR. The gel ran at 120V using 15 μL of DNA per well. Positive controls for the PCR were samples that had previously tested positive. The negative control for all reactions was nuclease‐free water. A negative result was defined as having detectable bacterial 16S rDNA PCR product from the same sample where no *wsp* product was detected. Both the 16S and *wsp* primers were used on all samples for accuracy.

### 2.3 Database

We added our field collections to a larger database from Weinert et al. (2015), along with two additional data sets of aquatic taxa (Sontowski et al., 2015; Wiwatanaratanabutr & Zhang, 2016). Insects were classified as aquatic or terrestrial based on their larval habitat. The full database is available in Appendix.

### 2.4 Model

We followed the previous literature by using a beta‐binomial model to estimate the incidence of *Wolbachia* (Hilgenboecker et al., 2008; Weinert et al., 2015; Zug & Hammerstein, 2012). In this model, incidence (proportion of individuals infected in a species or population) is described by a binomial distribution, and the distribution of incidences is described by a beta distribution. We estimated the posterior distribution of incidences using the following model:

(1) $$\begin{array}{cc} I_{i} & ∼\text{BetaBinomial}(n_{i},\;{\overset{¯}{p}}_{i},\;\theta )\\ & \text{logit}({\overset{¯}{p}}_{i})=\alpha +\beta x\\ \alpha & ∼\text{Normal}(0,3)\\ \beta & ∼\text{Normal}(0,3)\\ \theta & ∼\text{Exponential}(1) \end{array}$$

where *I*_*i*_ is the number of infected individuals in population *i*,* n*_*i*_ is the total number of individuals tested in population *i*, ${\overset{¯}{p}}_{i}$ is incidence, and θ is the shape parameter that describes the spread of the distribution. Numbers in parentheses indicate prior information about the parameters (e.g., *Normal* (0,3) means that the parameter comes from normal distribution with a mean of zero and standard deviation of 3). These are wide priors, so most inference comes from the data, not the priors. The model above generates a posterior distribution for θ*,* α, and β. From the posterior distribution, we estimated the mean and quantiles of θ and of $\text{logit}({\overset{¯}{p}}_{i})$ by solving the equation for both aquatic (where x = 0) and terrestrial (where *x* = 1) insects. We back‐transformed $\text{logit}({\overset{¯}{p}}_{i})$ to the probability scale using the logistic transformation. The resulting estimates for ${\overset{¯}{p}}_{i}$ and θ were then used to estimate the shape of the beta distribution.

From the beta distribution, we estimated the proportion of species infected with *Wolbachia* (incidence) by calculating the area under the curve of the beta distribution (mean distribution from 8,000 posterior estimates) that was >0.001. This is consistent with previous literature (Hilgenboecker et al., 2008; Weinert et al., 2015; Zug & Hammerstein, 2012) and means that a species is considered infected if at least 1/1,000 individuals carry *Wolbachia*. The proportion of the area under the curve that is >0.001 is equivalent to the proportion of species infected with *Wolbachia* (Hilgenboecker et al., 2008; Weinert et al., 2015; Zug & Hammerstein, 2012). To ensure that our model specifications were correct, we attempted to recreate previously published estimates of symbiont incidence for all arthropods based on the database of Weinert et al. (2015). Our model accurately reproduced their results (Fig. S1). In addition to estimating incidence among all aquatic and terrestrial insect species, we also estimated incidence separately for individual aquatic insect orders using the same model as above but without the term for β.

The full database may contain collection biases that could affect estimates of incidence (Weinert et al., 2015). First, some species are represented multiple times with others represented only once. This may reflect the fact that some studies target specific insects to test rather than randomly sampling insects, thereby biasing the dataset toward insects that have already tested positive for *Wolbachia*. Second, orders with few species may be overrepresented if species in those orders are targeted for the reason described above. To determine how these biases affected our conclusions, we ran analyses on two different databases containing (1) all samples from the global database (hereafter, “full database”) and (2) samples from the global database in which each species is represented only once (hereafter, “reduced database”). For the reduced database, we retained only the samples with the largest number of individuals tested in each species, following Weinert et al. (2015).

In addition to overrepresentation of species, we tested whether some orders were also overrepresented, potentially due to targeted sampling of species within particular orders. To test this, we plotted the number of species tested in each order against the total number of species in each order, as determined from Zhang (2013). There was a positive linear relationship (*r* ^2^=.84, Fig. S2), indicating no evidence of bias among orders. However, within aquatic insects, there was a clear overrepresentation of mosquitoes (Culicidae), almost certainly due to the importance of mosquitoes in disease transmission (Gubler, 1998). Culicidae made up 28% of the samples for all aquatic insects. To determine their effect on the results, we deleted mosquitoes from the database and reran the models above. We report results both with and without mosquitoes.

We generated posterior distributions for each parameter via the Hamiltonian Monte Carlo method using *rstan* (Stan Development Team 2016) via the *rethinking* package (McElreath, 2016) in R. For each model, we ran four independent chains for 2,000 iterations, generating 8,000 total estimates of the posterior distribution for each parameter. We assessed convergence visually using trace plots and by ensuring that r‐hat (potential scale reduction factor) was <1.1. All models achieved convergence. The full database is available in Appendices S1, S2, along with R scripts for each model. Results of each model, including trace plot diagnostics, are available in the Supplementary Information (Table S2).

## 3 Results

**Figure 1:**
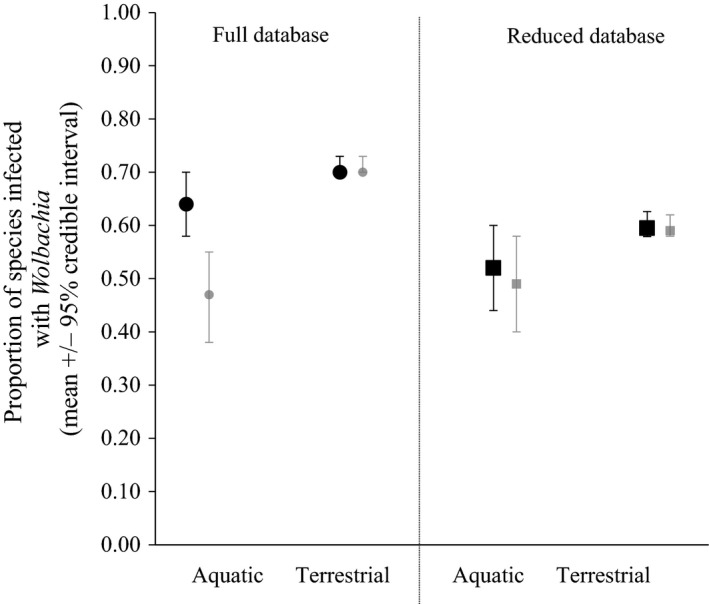
Mean (±95% credible intervals) of *Wolbachia* incidence in aquatic versus terrestrial insects. Circles represent estimates from the full dataset. Squares represent estimates from the reduced database retaining only one sample per species (sample with the maximum number of screens). Black symbols include Culicidae. Gray symbols do not

We estimate that 52% (CrIs: 44%–60%) (mean [95% credible intervals]) of aquatic insect species are infected with *Wolbachia* versus 60% (58%–63%) of terrestrial insects. These estimates come from the reduced database in which only one sample per species is included. The need for this reduced database arises because estimates from the full database may have been affected by targeted sampling of species known to have *Wolbachia*. Additionally, the removal of Culicidae from the reduced database does not appreciably alter our estimate of incidence in aquatic insects (52% vs. 49%) (Figure 1). Estimates from the full database show 64% (58%–70%) of aquatic insect species are infected with *Wolbachia* versus 70% (70%–73%) of terrestrial insects (Figure 1). The exclusion of Culicidae reduced the estimate of incidence for aquatic insects in the full database from 64% to 47% (Figure 1).

**Figure 2:**
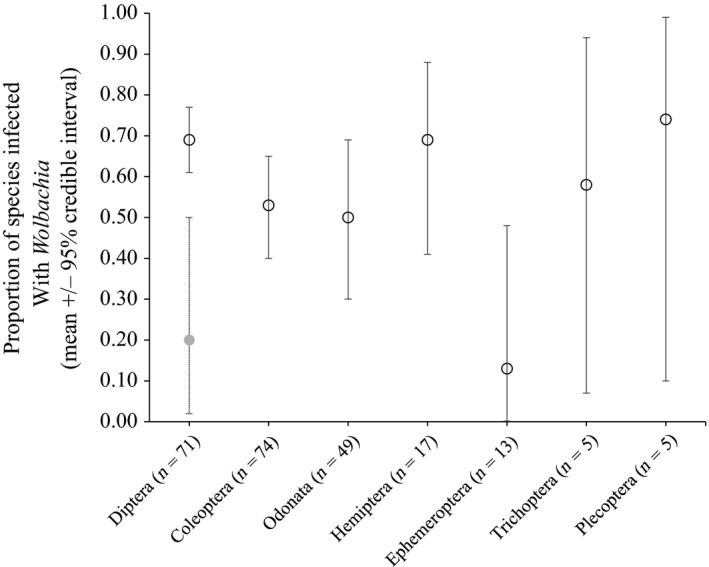
Incidence of *Wolbachia* among aquatic insect orders. Data are the mean and 95% credible intervals derived from a model with all samples for each order. The *x*‐axis notes the order and the number of species sampled in each order (in parentheses). The gray circle represents an estimate of incidence without Culicidae. See the Supplementary Information (Fig. S3) for a comparison between this analysis and results with the reduced dataset (i.e., each species represented only once). Samples are sorted in order of increasing uncertainty of the estimates

To estimate incidence within aquatic orders, we ran separate models for each order. We did this for both the full database and reduced database but present results only for the full database, since they are similar to those using the reduced database (Fig. S3). Among aquatic insects, Diptera (69% (61%–77%)) and Hemiptera (69% (41–88)) had the highest incidence (Figure 2). However, high incidence in Diptera was due to the overrepresentation of Culicidae. When they were removed, the estimate of incidence in Diptera dropped to 20% with wide credible intervals (2%–50%). Coleoptera (53% (40–65)) and Odonata (50% (30–69)) had the next highest rates of incidence while Ephemeroptera (13% (0–48)) had the lowest (Figure 2). Trichoptera and Plecoptera had low sample sizes (4 and 5 species in each order, respectively). As a result, 95% credible intervals for Trichoptera (7%–94%) and Plecoptera (10%–99%) spanned nearly the entire distribution of incidence rates, indicating very little certainty in incidence within these groups. Megaloptera was included in our database as just a single sample (negative test for *Wolbachia*), so no estimate is provided for this order.

## 4 Discussion

*Wolbachia* are common intracellular bacteria in aquatic insects with an estimated incidence among species of 52% compared to 60% for terrestrial insects. Taken together, these results are similar to the previous estimates of incidence within all arthropods of 40%, 52%, and 66% (Hilgenboecker et al., 2008; Weinert et al., 2015; Zug & Hammerstein, 2012). Despite clear evolutionary and ecological separation between aquatic and terrestrial insects, it is now clear from these data that *Wolbachia* infection is potentially common in aquatic ecosystems.

Our results represent the first estimates of incidence in common aquatic insect orders (Ephemeroptera, Trichoptera, Plecoptera). Most studies investigating aquatic insects have primarily focused on mosquitoes, including a recent study where 67% of samples were mosquitoes (Wiwatanaratanabutr & Zhang, 2016). This focus is likely due to the role that mosquitoes play as important vectors for disease, making them an important target for sampling. Estimates from other surveys appear to mostly include aquatic insects only haphazardly during sampling of terrestrial insects (but see Sontowski et al., 2015). Of the aquatic insects tested in previous studies*,* most belonged to Odonata, Coleoptera, or Diptera (Hilgenboecker et al., 2008; Weinert et al., 2015; Zug & Hammerstein, 2012). As a result of this targeted collection, our estimates of incidence in Odonata, Coleoptera, and Diptera have less uncertainty than estimates from Ephemeroptera, Trichoptera, and Plecoptera. There is clearly an opportunity to increase sample size among these orders, particularly given their importance as indicators of ecosystem health. For example, EPT indices are used worldwide as a proxy for stream water quality (Carter, Resh, Hannaford, & Myers, 2006). Understanding the incidence and effects of symbionts on these taxa, and their potential interaction with contaminants, may reveal important, but understudied, impacts of symbionts on water quality indicators.

Knowing the distribution of *Wolbachia* in all insect taxa is important to understand the risks of using *Wolbachia* as a biocontrol. Since mosquitoes spend a portion of their life in an aquatic environment and return to the water to lay eggs, the insects most likely to be affected by an introduction of infected mosquitoes would be those that either eat mosquitoes or live in the same environment as them. One major concern is a lack of research into horizontal transfer and regulation (Loreto & Wallau, 2016). Given the complexities and risks of biocontrol, it is important to weigh the costs and benefits of introducing *Wolbachia* into novel populations (Ahmed, Li et al., 2015). Our results shed light on this risk by revealing that *Wolbachia* infection is common among aquatic insects. As a result, introducing an infection within one species, especially an invasive species, may not pose a serious risk (Dobson, Bordenstein, & Rose, 2016). However, different strains of *Wolbachia* will pose different risks to the hosts (Hoffmann, Ross, & Rašić, 2015; Ritchie, Townsend, Paton, Callahan, & Hoffmann, 2015). Releasing a virulent strain into the environment that can interfere with reproduction with the intended consequence of reducing abundance of one insect species may carry unintended consequences for nontarget taxa. Moreover, it is unclear how consistent incidence is among sites. While our estimates represent a global mean incidence, it seems clear that incidence and prevalence within populations is certain to vary widely (Ahmed, Araujo‐Jnr, Welch, & Kawahara, 2015). That in turn would cause spatial variation in the risk of *Wolbachia* spreading to nontarget populations.

*Wolbachia* can affect insects at the population scale by altering sex ratios and population sizes (Werren & Buekeboom, 1998; Mains et al. 2013). However, to our knowledge, the effects of *Wolbachia* at the community or ecosystem level have not been addressed. Given that *Wolbachia* is common in aquatic insect species and its potential to alter population sizes (e.g., Mains et al. 2013), there is a strong need to understand how its effects scale up to potentially alter ecosystem functions provided by aquatic insects. These include secondary production in freshwater ecosystems and the subsequent flux of insect biomass from aquatic to terrestrial ecosystems (Nakano & Murakami, 2001).

We estimate that approximately 52% of aquatic insect species have at least one individual infected with *Wolbachia*. These results show that *Wolbachia* is present in aquatic insects at a similar incidence seen in terrestrial insects. With the push toward using *Wolbachia* as a biocontrol (Yakob & Walker, 2016), future work should focus on understanding how these bacteria influence their hosts and the ecosystem services that aquatic insects provide. Future research should also focus on narrowing the uncertainty of the numbers attained in this report by more comprehensive sampling of the infected areas, including sampling terrestrial insects from the same areas. Genotyping of *Wolbachia* strains using methodology from Baldo et al. (2006) should also provide clues of how *Wolbachia* is spatially distributed.

## Conflict of interest

None declared.

## Data accessibility

Data are located in the *Knowledge Network for Biocomplexity* database. Appendix 1 is located at doi:10.5063/F1HX19N1. Appendix 2 is located at doi:10.5063/F1NP22D6. The R code for the model can be found at https://github.com/ericjsazama/IncidenceofWolbachia.
