# Supplementary material for: Incidence of Wolbachia in aquatic insects
Source: Ecol Evol. 2017 Jan 24;7(4):1165–9. doi: 10.1002/ece3.2742 (PMC5306009; doi:10.1002/ece3.2742)
Supplement: Supplementary file 1 [file ECE3-7-1165-s001.docx]

Supplementary Information for Sazama et al. Incidence of *Wolbachia* in aquatic and terrestrial insects

Sampling Sites

| Table S1. GPS coordinates for the sampling sites used in this study. Rivers where multiple sites were sampled are indicated with different numbers. | | |
| --- | --- | --- |
| Sampling Locations | | |
| River | Latitude | Longitude |
| Baptist Creek | 42° 54' 36.22" | 96° 54' 52.94" |
| Bazille Creek | 42° 45' 23.07" | 97° 56' 47.96" |
| Big Sioux River | 42° 39' 42.38" | 96° 32' 39.91" |
| Bow Creek | 42° 43' 42.57" | 97° 8' 48.35" |
| ExARS | 42° 48' 1.79" | 96° 55' 36.37" |
| James River | 42° 52' 49.59" | 97° 16' 44.07" |
| Missouri River 1 | 42° 45' 59.90" | 97° 0' 18.10" |
| Missouri River 2 | 42° 45' 36.71" | 96° 58'10.28" |
| Missouri River 3 | 42° 44' 50.34" | 96° 57' 11.11" |
| Niobrara River | 42° 44' 54.16" | 98° 3' 25.91" |
| Spirit Mound Creek | 42° 52' 6.78" | 96° 57' 18.21" |
| Verdigre Creek | 42° 36' 20.49" | 98° 2' 4.04" |
| Vermillion River 1 | 42° 54' 35.76" | 96° 54' 58.25" |
| Vermillion River 2 | 43° 5' 22.43" | 96° 57' 46.99" |

Model verification

To ensure that our modelling procedure was consistent with previous estimates of symbiont incidence, we modeled incidence for *Wolbachia, Rickettsia,* and *Cardinium* using the database of Weinert et al. (2015). Their estimates focused on incidence of these symbionts among all arthropods. We compared their results, as estimated visually from Figure 1 in Weinert et al. (2015), to our own estimates based on the Bayesian model presented in the main text and reproduced here:

*I_i_ ~ BetaBinomial*(*n_i_****, p̄****_i_*, *θ*)

logit(***p̄****_i_*) = α

α ~ *Normal* (0,3)

*θ ~ Exponential (1)*

where *I_i_* is the number of infected individuals in population *i*, *n_i_* is the total number of individuals tested in population *i*, ***p̄****_i_* is prevalence, and *θ* is the shape parameter that describes the spread of the distribution. Numbers in parentheses indicate prior information about the parameters (e.g. *Normal* (0,3) means that the parameter comes from normal distribution with a mean of zero and standard deviation of 3). The parameters in this model are the intercept, α, which estimates logit(***p̄****_i_* ), and theta, *θ*, which estimates the spread of the distribution. We use theta, along with the back-transformed ***p̄****_i_* (via the logistic transformation), to estimate the beta distribution. From this distribution, we estimate the proportion of samples with prevalence > 0.001.

Figure S1 shows the performance of our model relative to estimates in Weinert et al. 2015). Estimates of incidence for each symbiont are nearly identical to those of Weinert et al. (2015), which itself is based on Hilgenboecker et al. (2008), indicating that our model is consistent with previous efforts to estimate incidence of *Wolbachia* and other symbionts in arthropods.

Figure S1. Recreation of incidence estimates using our Bayesian model (Sazama) in comparison to estimates from Weinert et al. (2015). Estimates represent the mean incidence and either 95% confidence intervals (Weinert) or 95% credible intervals.


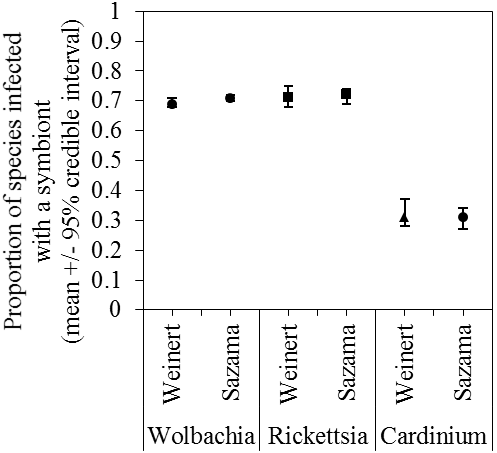


Bias of rare orders

To determine whether some orders were overrepresented in the database relative to the number of species in those orders, we plotted the natural log of the number of species in each order against the natural log of the number of species sampled in database within each order. There was strong positive relationship, indicating no discernable bias.

Figure S2. Relationship between the natural log of the number of species in each order, and the natural log of the number of species tested in each order.


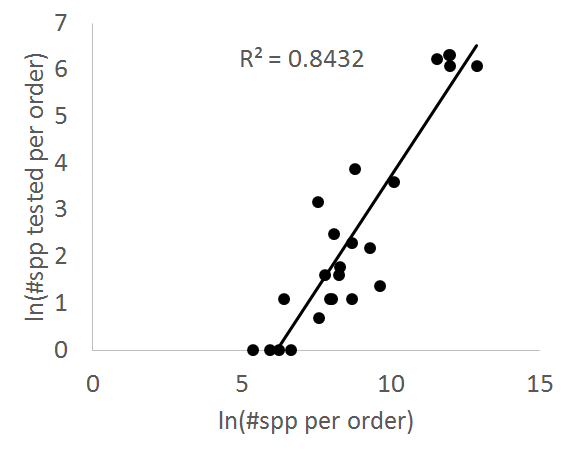


Full results of each model

| Table S2. Model results for each model in the manuscript. "theta" is a shape parameter that describes the spread of the distribution, "a" is the intercept, and "b" is the difference between aquatic and terrestrial insects, where b*0 estimates prevalence terrestrial insects (i.e. intercept only) and b*1 estimates prevalence for aquatic insects. These parameter values were used to estimate the beta distribution, from which estimates of incidence were derived. n_eff is the number of effective samples out of 8000 posterior estimates. R_hat is the Gelman-Rubin convergence diagnostic (Gelman and Rubin 1992), where values ~1 indicate that all four chains converged. See the full R script in the Supplementary Material. | | | | | | | |
| --- | --- | --- | --- | --- | --- | --- | --- |
| Aquatic versus terrestrial insects | | | | | | | |
|  |  | Mean | StdDev | lower 95% | upper 95% | n_eff | R_hat |
| *Full database* | |  |  |  |  |  |  |
|  | theta | 0.41 | 0.02 | 0.38 | 0.43 | 5457 | 1 |
|  | a | -0.74 | 0.03 | -0.8 | -0.69 | 5566 | 1 |
|  | b | -0.25 | 0.11 | -0.47 | -0.02 | 4840 | 1 |
| *Full database - no mosquitoes (Culicidae)* | | | | |  |  |  |
|  | theta | 0.42 | 0.02 | 0.38 | 0.45 | 6274 | 1 |
|  | a | -0.75 | 0.03 | -0.8 | -0.7 | 6565 | 1 |
|  | b | -0.88 | 0.16 | -1.2 | -0.59 | 6018 | 1 |
| *Reduced database* | | |  |  |  |  |  |
|  | theta | 0.39 | 0.03 | 0.33 | 0.44 | 7188 | 1 |
|  | a | -1.09 | 0.04 | -1.17 | -1.01 | 6033 | 1 |
|  | b | -0.27 | 0.14 | -0.55 | 0.01 | 6581 | 1 |
| *Reduced database - no mosquitoes (Culicidae)* | | | | |  |  |  |
|  | theta | 0.39 | 0.03 | 0.34 | 0.45 | 5870 | 1 |
|  | a | -1.11 | 0.04 | -1.19 | -1.03 | 5680 | 1 |
|  | b | -0.39 | 0.16 | -0.7 | -0.07 | 6040 | 1 |
| Individual aquatic orders | | | | | | | |
|  |  | Mean | StdDev | lower 95% | upper 95% | n_eff | R_hat |
| *Diptera only* | |  |  |  |  |  |  |
|  | theta | 0.27 | 0.05 | 0.17 | 0.38 | 5857 | 1 |
|  | a | -0.47 | 0.17 | -0.81 | -0.15 | 5731 | 1 |
| *Diptera only - no mosquitoes (Culicidae)* | | | | |  |  |  |
|  | theta | 1.54 | 1.19 | 0.02 | 3.84 | 4911 | 1 |
|  | a | -4.05 | 0.92 | -5.9 | -2.39 | 3758 | 1 |
| *Coleoptera only* | | |  |  |  |  |  |
|  | theta | 0.13 | 0.14 | 0 | 0.41 | 4656 | 1 |
|  | a | -0.58 | 0.23 | -1.03 | -0.12 | 4767 | 1 |
| *Hemiptera only* | | |  |  |  |  |  |
|  | theta | 0.68 | 0.32 | 0.16 | 1.31 | 5266 | 1 |
|  | a | -1.32 | 0.43 | -2.2 | -0.49 | 4533 | 1 |
| *Plecoptera only* | | |  |  |  |  |  |
|  | theta | 1.38 | 1.03 | 0.02 | 3.43 | 5519 | 1 |
|  | a | -2.1 | 1.03 | -4.21 | -0.17 | 4621 | 1 |
| *Trichoptera only* | | |  |  |  |  |  |
|  | theta | 1.39 | 1.09 | 0.01 | 3.53 | 4104 | 1 |
|  | a | -2.61 | 1 | -4.71 | -0.85 | 3483 | 1 |
| *Odonata only* | |  |  |  |  |  |  |
|  | theta | 1.01 | 0.35 | 0.41 | 1.71 | 5123 | 1 |
|  | a | -2.27 | 0.32 | -2.87 | -1.6 | 4651 | 1 |
| *Ephemeroptera only* | | |  |  |  |  |  |
|  | theta | 0.86 | 0.89 | 0 | 2.7 | 5247 | 1 |
|  | a | -4.55 | 1.52 | -7.59 | -1.77 | 3398 | 1 |

Comparison of incidence among orders using full and reduced datasets

Figure S3. Comparison of incidence estimates for each order using the full dataset (circles) or the reduced dataset (red bars). Credible intervals for the reduced dataset are not shown for clarity. Comparisons are only made for orders in which the full and reduced datasets differed (i.e. there were no duplicate species in the Coloeptera, Trichoptera, or Plecoptera orders). Numbers in parentheses on the x-axis represent the number of species sampled.
